# Pulmonary Benign Metastasizing Leiomyoma: A Retrospective Analysis of Seven Cases Including a Rare Coexistence with In Situ Mucinous Adenocarcinoma

**DOI:** 10.3390/biomedicines13081971

**Published:** 2025-08-13

**Authors:** Zeguang Ye, Xi Wu, Can Fang, Min Zhu

**Affiliations:** Department of Thoracic Surgery, Tongji Hospital, Tongji Medical College, Huazhong University of Science and Technology, Wuhan 430030, China

**Keywords:** pulmonary benign metastasizing leiomyoma (PBML), uterine leiomyoma, in situ mucinous adenocarcinoma, immunohistochemistry

## Abstract

**Background:** Pulmonary benign metastasizing leiomyoma (PBML) is a rare condition characterized by histologically benign smooth muscle tumors occurring at extrauterine sites, often in women with a history of uterine leiomyoma. While PBML generally exhibits indolent behavior, its pathogenesis, management, and malignant potential remain unclear. **Methods:** This study retrospectively analyzes the clinical characteristics, imaging features, diagnostic approaches, pathological findings, treatment strategies, and outcomes of seven patients with PBML treated at our institution between January 2016 and May 2025. **Results:** Seven patients were included, with a mean age at diagnosis of 48.9 ± 5.6 years. Two patients presented with respiratory symptoms. Imaging revealed multiple bilateral pulmonary nodules in four patients and solitary nodules in three. Six patients were diagnosed via video-assisted thoracoscopic surgery, and one through computed tomography-guided percutaneous biopsy. Immunohistochemistry revealed positivity for SMA and Desmin in all cases, ER in six, and PR in five, with the Ki-67 labeling index ≤3% in six patients. One patient had a coexisting in situ mucinous adenocarcinoma within the PBML lesion. All had a history of uterine leiomyoma. After diagnosis, one patient received hormonal therapy, and another underwent right adnexectomy. The remaining patients were managed with surveillance without additional treatment. During follow-up, one patient developed distant organ metastasis. **Conclusions:** PBML is a rare, typically indolent condition with potential for metastasis. Accurate diagnosis relies on imaging, histopathology, and immunohistochemistry. This study reports a unique case of PBML coexisting with intratumoral in situ mucinous adenocarcinoma, a previously unreported finding that may broaden the known histopathological spectrum.

## 1. Introduction

Benign metastasizing leiomyoma (BML), first described by Steiner in 1939, is a rare condition characterized by the presence of histologically benign smooth muscle tumors in extrauterine sites [1]. It typically affects women of reproductive or perimenopausal age and is often associated with a history of uterine leiomyomas, many of whom have undergone prior hysterectomy or myomectomy [2].

Pulmonary benign metastasizing leiomyoma (PBML) is the most frequent manifestation of BML and is usually discovered incidentally during imaging studies, as many patients remain asymptomatic [2,3,4,5]. Despite its benign histological appearance, BML exhibits metastatic behavior, which challenges traditional distinctions between benign and malignant tumors [6,7,8]. The pathogenesis of BML remains unclear. The majority of BML lesions express estrogen receptor (ER) and progesterone receptor (PR), suggesting that hormonal factors may play a role in tumor progression and potentially guide treatment options [9,10]. Due to the rarity of this condition, there is no standardized diagnostic or therapeutic approach. Diagnosis often requires histopathological confirmation through surgical resection or biopsy, supported by immunohistochemical markers such as smooth muscle actin (SMA), Desmin, Caldesmon, ER, and PR [6,9,11]. The clinical course is typically indolent; however, in rare cases, distant metastases or atypical behavior may occur, necessitating long-term surveillance [7,8].

In this study, we present a retrospective analysis of seven cases of PBML diagnosed at our institution over a nine-year period. Notably, we report the first known case of PBML coexisting with intratumoral in situ mucinous adenocarcinoma, a previously unreported finding. This highlights the importance of thorough pathological assessment and adds new insight into the clinical spectrum of PBML.

## 2. Materials and Methods

### 2.1. Study Design and Patients

This retrospective case series included all patients diagnosed with PBML at Tongji Hospital, Tongji Medical College, Huazhong University of Science and Technology between January 2016 and May 2025. All cases were pathologically confirmed. Inclusion criteria were (1) a diagnosis of PBML based on pathological examination following either video-assisted thoracoscopic surgery (VATS) or computed tomography (CT)-guided percutaneous biopsy (CTPB); (2) continuous follow-up during the study period. Exclusion criteria included inability to complete follow-up or unknown clinical outcomes during the study timeframe. This study was approved by the Ethics Committee of Tongji Hospital, Tongji Medical College, Huazhong University of Science and Technology (Approval No. TJ-IRB202412136).

### 2.2. Data Collection

Clinical and follow-up data were obtained from the hospital’s electronic medical record system and through direct telephone contact with patients or their families. Collected variables included demographic and clinical characteristics (age at diagnosis, reproductive history, presenting symptoms, duration of active surveillance, and gynecologic history), radiological features (tumor size, morphology, location, and whether lesions were solitary or multiple based on chest CT), treatment details, pathological findings, and postoperative adjuvant therapies. As this was a retrospective study, not all immunohistochemical markers were assessed in every patient.

Follow-up was conducted through 1 May 2025. Clinical status, including recurrence or evidence of metastasis, was evaluated at each follow-up. Patients without clinical events were followed until the last contact date, while follow-up was terminated upon disease progression, recurrence, or death.

### 2.3. Statistical Analysis

Descriptive statistics were used to summarize the clinical, radiological, pathological, and treatment-related data. Continuous variables are presented as mean ± standard deviations, while categorical variables are expressed as numbers and percentages. Given the small sample size and the exploratory nature of this retrospective case series, no inferential statistical tests were performed. All data analyses were conducted using SPSS 27.0 (IBM Corp., Armonk, NY, USA).

## 3. Results

### 3.1. Characteristics of the Patients

A total of seven female patients diagnosed with PBML were included in this retrospective case series. The mean age at diagnosis was 48.9 ± 5.6 years (range: 40–58 years). Among them, two patients (28.6%) presented with symptoms of cough and sputum production, while the remaining five (71.4%) were asymptomatic and diagnosed incidentally during routine physical examinations. All seven patients had a documented history of uterine leiomyoma. Five patients (71.4%) had previously undergone surgical treatment for uterine leiomyoma, while two (28.6%) had not received any surgical intervention (Table 1).

### 3.2. Radiological Imaging Characteristics

**Figure 1:**
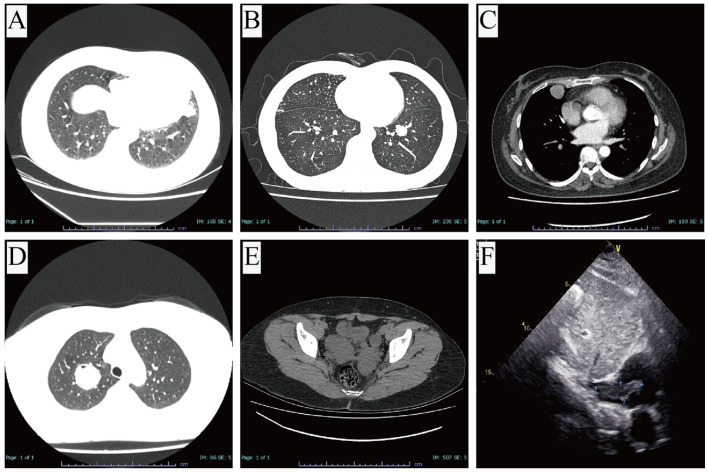
Imaging findings: (**A**–**D**) chest computed tomography images of Case 1, Case 4, Case 5, and Case 7, respectively, showing pulmonary nodules in different locations; (**E**,**F**) Case 1’s pelvic computed tomography and transthoracic echocardiogram three years after surgery.

Contrast-enhanced chest CT revealed multiple bilateral pulmonary nodules in four (57.1%) patients and solitary solid nodules in three (42.9%). All nodules demonstrated well-defined margins and relatively homogeneous attenuation. Post-contrast images showed no or only mild enhancement. The nodules were predominantly solid in nature, with the exception of one lesion in Case 7, which appeared as a partially cystic-solid mass on imaging. However, an earlier CT scan performed at an outside hospital had shown a solid nodule prior to CTPB. Given this, we considered the cystic component in Case 7 to be a result of post-biopsy changes. The mean maximum diameter of the pulmonary lesions was 22.4 ± 13.4 mm (range: 9–46 mm), with the largest observed in Case 7 (Figure 1A–D). Positron emission tomography–computed tomography (PET/CT) was performed in Cases 6 and 7 (28.6%). In these two cases, the pulmonary lesions demonstrated no significant or only mildly increased metabolic activity. The largest nodules in each patient exhibited mildly elevated fluorodeoxyglucose uptake, with SUVmax values of 1.9 and 2.3, respectively. Notably, PET/CT findings in Case 7 suggested the possibility of a parasitic infection or a low-grade malignancy, reflecting the diagnostic uncertainty in such cases.

The duration of active surveillance prior to definitive diagnosis ranged from 1 week to 5 years. During this period, gradual enlargement of pulmonary nodules was observed in Cases 1 and 5. Case 5, who underwent the longest period of surveillance, demonstrated an increase in nodule size from 19 mm to 30 mm over five years.

### 3.3. Surgical Procedures and Pathological Findings

Among the seven patients included in this series, Case 6 (14.3%) was diagnosed with PBML via CTPB, while Case 7 underwent the same procedure at an outside hospital but without a definitive diagnosis. Therefore, including Case 7, six patients (85.7%) ultimately underwent VATS, including lobectomy or wedge resection, for pathological confirmation.

Intraoperative frozen section analysis was performed in five out of the six thoracoscopic cases, excluding Case 7. Among these, Case 2 was initially suspected to be a sclerosing pneumocytoma, while the remaining four were interpreted as pulmonary spindle cell lesions of uncertain nature.

**Figure 2:**
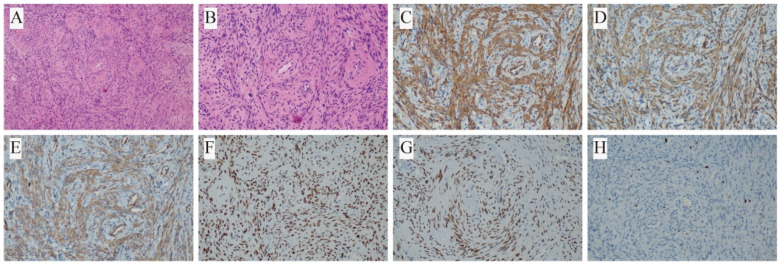
Histopathological and immunohistochemical features of the leiomyoma component in Case 7: (**A**,**B**) H&E staining at 100× and 200× magnification; (**C**) positive for SMA; (**D**) positive for Desmin; (**E**) positive for Caldesmon; (**F**) positive for ER; (**G**) positive for PR; (**H**) Ki-67 labeling index approximately 2%.

**Figure 3:**
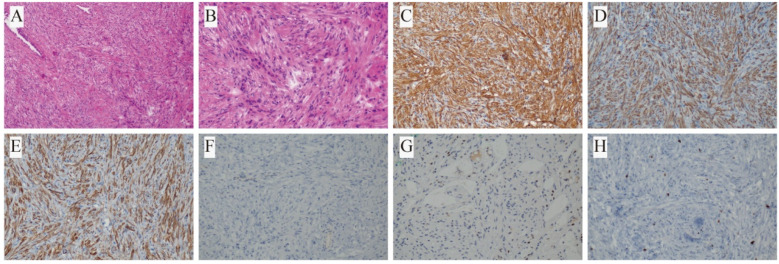
Histopathological and immunohistochemical features of the leiomyoma component in Case 5: (**A**,**B**) hematoxylin and eosin (H&E) staining at 100× and 200× magnification; (**C**) positive staining for SMA; (**D**) positive for Desmin; (**E**) positive for Caldesmon; (**F**) negative ER; (**G**) weakly positive PR; (**H**) Ki-67 labeling index approximately 3%.

Subsequent permanent pathological evaluation revealed that all lesions were of smooth muscle origin, consistent with a diagnosis of PBML. Immunohistochemical analysis was performed in all seven patients (Table 2). All cases showed positive staining for SMA and Desmin. Caldesmon was positive in five cases (71.4%), with two cases (28.6%) not tested. Vimentin (VIM) was positive in two cases (28.6%) and not assessed in the remaining five (71.4%). ER was positive in five cases (71.4%), weakly positive in one (14.3%), and negative in one (14.3%). PR was positive in four cases (57.1%), weakly positive in one (14.3%), and not tested in two (28.6%). The Ki-67 labeling index (LI) was ≤3% in six cases (85.6%), indicating low proliferative activity (Figure 2 and Figure 3). In contrast, Case 2 (14.3%) showed a Ki-67 LI of approximately 10%. However, fluorescence in situ hybridization (FISH) for the JAZF1 gene rearrangement was negative, which helped exclude the possibility of metastatic low-grade endometrial stromal sarcoma.

**Figure 4:**
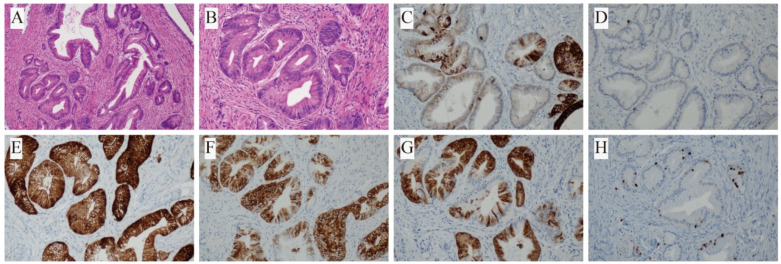
Histopathological and immunohistochemical features of the glandular component in Case 5: (**A**,**B**) H&E staining at 100× and 200× magnification; (**C**) partial positivity for CK20; (**D**) scattered positivity for CDX2; (**E**) positive for Villin; (**F**) positive for MUC5AC; (**G**) positive for MUC6; (**H**) Ki-67 labeling index approximately 10%.

A particularly rare finding was noted in Case 5. Within the PBML lesion, invaginated alveolar epithelium was identified, and focal areas exhibited transformation into mucinous epithelium with papillary and micropapillary proliferative patterns. These epithelial components displayed a gastrointestinal immunophenotype and low-grade cytologic atypia. The immunohistochemical profile of this component was as follows: CK20 (focal +), CDX2 (scattered +), Villin (+), MUC5AC (+), MUC6 (+), TTF-1 (−), P63 (−), P53 (scattered +, wild-type pattern), and Ki-67 LI ~10% (Figure 4). Comprehensive preoperative evaluations revealed no abnormalities in other organs, effectively excluding the possibility of metastasis. Based on the morphological and immunohistochemical findings, the final diagnosis was considered to be a PBML coexisting with intratumoral in situ mucinous adenocarcinoma, an exceedingly rare occurrence not previously reported to our knowledge.

### 3.4. Adjuvant Treatment and Follow-Up

Case 1 had previously undergone a total hysterectomy and right adnexectomy for a uterine leiomyoma. Two months following the thoracoscopic lung resection, she underwent a left adnexectomy. No additional adjuvant therapy was administered. She remained under regular surveillance at our institution. Unfortunately, three years after VATS, routine follow-up revealed new masses in the pelvic cavity and inferior vena cava (Figure 1E,F). Surgical resection was performed, and histopathology confirmed the diagnosis of BML. Case 6 received gonadotropin-releasing hormone (GnRH) analog therapy following CTPB. Given the unique coexistence of in situ mucinous adenocarcinoma within the PBML lesion in Case 5, a multidisciplinary team (MDT) consultation was conducted postoperatively, involving thoracic surgery, oncology, gynecology, and pathology specialists. Taking into account the patient’s own preferences, it was ultimately decided not to administer adjuvant therapy but to conduct regular and structured follow-up through the MDT clinic to avoid overlooking any potential recurrence or metastasis. The remaining four patients also opted for routine surveillance without adjuvant therapy following VATS.

As of the last follow-up, with a mean follow-up time of 26.0 ± 12.3 months (range: 14–44 months), no evidence of recurrence or metastasis was observed in any patient except Case 1. All six (85.7%) recurrence-free patients will continue routine imaging surveillance to monitor for potential disease progression.

## 4. Discussion

BML, a rare disease first reported by Steiner in 1939, is a benign tumor that typically develops in premenopausal women with a history of uterine leiomyoma [1,2]. As the most common manifestation of BML, PBML is typically detected months to years after hysterectomy or myomectomy and generally exhibits slow growth [3]. Barnaś et al. [12] conducted a literature review using Medline/PubMed, Embase, Web of Science, and Cochrane databases and reported that the mean age of patients at the time of BML diagnosis was 47.3 years, while the mean age at the time of uterine surgery was 38.5 years. According to Kayser et al. [11], the average interval between hysterectomy and the development of pulmonary lesions is 14.9 years, and neither the location nor the number of metastases is influenced by this interval [12]. In our retrospective case series, the mean age at diagnosis among the seven PBML patients was 48.9 ± 5.6 years (range: 40–58 years). According to the literature, approximately 19 cases of PBML without prior uterine leiomyoma surgery have been reported [6,12]. In our study, two patients (28.6%) had not undergone any related gynecological surgery before diagnosis. Among the five patients who had undergone surgery for uterine leiomyoma, the mean age at the time of surgery was 35.7 ± 7.6 years (range: 26–44 years). These findings show slight differences compared with previous reports, which may be attributed to variations in sample size and ethnic background. Although uterine leiomyomas are more prevalent among Black women [13], most reported cases of PBML lack information on patients’ racial backgrounds. Further research is warranted to investigate whether this epidemiologic disparity translates to differences in PBML incidence or clinical characteristics.

PBML is an exceptionally rare disease defined by a distinctive combination of benign pathological characteristics and a tendency for tumor-like metastasis with malignant potential [14,15]. To date, fewer than 200 cases have been documented in the literature [4]. A few reports have described the coexistence of PBML and primary malignant pulmonary tumors occurring at distinct anatomical sites [16,17]. However, the presence of a malignant neoplasm arising within a PBML lesion itself is an even more exceedingly rare phenomenon.

### 4.1. Clinical Manifestations and Pathogenesis

PBML typically exhibits indolent progression, with most patients remaining asymptomatic at diagnosis and lesions often incidentally detected through imaging studies [12,18,19,20]. Approximately one-third to one-half of PBML patients may present with respiratory symptoms such as cough, sputum production, hemoptysis, and dyspnea; in severe cases, respiratory failure may occur [12,18,19,21,22,23,24]. Hao et al. reviewed the clinical characteristics of 65 PBML patients reported in the literature, among whom 28 were asymptomatic, 20 had dyspnea, 10 had cough, 5 presented with pneumothorax, 3 had hemoptysis, 2 reported chest pain or tightness, and 1 experienced backache [25]. In our case series, two patients presented with clinical symptoms of cough and sputum production, while the remaining five were asymptomatic, with lesions detected incidentally during routine imaging examinations.

The pathogenesis of PBML remains incompletely elucidated and is a topic of controversy, with several prevailing hypotheses currently proposed [3,12,19,23,26]: 1. hematogenous and lymphatic transmission; 2. the true metastasis of low-grade leiomyosarcoma originating from the uterus; 3. inadvertent peritoneal seeding of fragments from uterine leiomyomas during hysterectomy or myomectomy; and 4. the metaplastic transformation of coelomic tissue. The first one is the most widely accepted [3,12].

A few reported cases have described the occurrence of PBML and primary malignant lung tumors in different pulmonary locations [16,17]. In addition, malignant transformation of PBML into leiomyosarcoma has also been reported [20,27,28]. Notably, our Case 5 represents a unique instance of in situ mucinous adenocarcinoma arising within a PBML lesion. Histologically, the two components were well demarcated yet spatially adjacent, separated by fibrous stroma. Immunohistochemically, the PBML region demonstrated classic smooth muscle markers (SMA+, Desmin+, and Caldesmon+), minimal hormone receptor expression (ER- and PR weak+), wild-type p53 (scattered positivity), and a low proliferative index (Ki-67 ~3%). In contrast, the glandular component expressed epithelial/mucinous markers (partial CK20+, focal CDX2+, Villin+, MUC5AC+, and MUC6+), lacked TTF-1, exhibited Ki-67 ~10%, and had similar wild-type p53. This histological juxtaposition and immunophenotypic distinction argue against a collision tumor and suggest a true coexistence.

Although the exact pathogenesis remains unclear, a few hypotheses may be considered. First, the possibility of a chance coexistence cannot be completely ruled out. Second, chronic hormonal stimulation or local microenvironmental factors might contribute to the transformation or emergence of neoplastic epithelial elements within a benign smooth muscle background. Analogous phenomena have been reported in endometriosis-associated malignancies [29,30]. The pathogenesis of endometriosis remains unclear. However, several hypotheses—such as coelomic metaplasia, vascular or lymphatic dissemination, and genetic predisposition—have been proposed, which are also similar to those suggested for the development of PBML [6,19,23,31]. High levels of inflammatory mediators and activated cytokines in the endometriotic microenvironment contribute to the recruitment and dysfunction of nearly all types of immune cells. This chronic inflammatory milieu promotes angiogenesis, cellular proliferation, invasion, and eventually tumor progression [29,31]. Furthermore, the persistent exposure to elevated levels of estrogen, a known proliferative and pro-survival hormone, is also considered a risk factor for malignant transformation [29]. Given the histogenetic and hormonal similarities, these mechanisms may offer insight into the rare coexistence of in situ mucinous adenocarcinoma within PBML, as observed in our case. Although PBML is typically regarded as a benign estrogen-sensitive smooth muscle proliferation, the presence of chronic hormone stimulation and local immune dysregulation could potentially create a microenvironment conducive to neoplastic transformation of entrapped or metaplastic epithelial elements.

Therefore, while no definitive causal link can be established, this case highlights the potential for malignant transformation or concurrent neoplastic processes within PBML lesions. Thorough sampling and careful histopathological evaluation are essential, especially when radiologic or intraoperative findings raise suspicion of heterogeneity. Further accumulation of similar cases is needed to clarify whether this coexistence represents a unique biological phenomenon or a fortuitous event.

### 4.2. Diagnosis and Differentiation

The diagnosis of PBML currently lacks specific methods and primarily relies on medical history and pathology [6,11]. PBML typically presents as well-defined nodules with various patterns on lung CT imaging [2]. These nodules can be solitary or diffusely distributed, appearing as isolated small nodules or masses [7,32].

Most PBML pulmonary nodules typically exhibit minimal or no metabolic uptake of fluorine-18 fluorodeoxyglucose (^18^F-FDG) activity [33,34]. Therefore, ^18^F-FDG PET/CT can be a useful tool in differentiating PBML from other malignancies [35,36]. The latest studies have demonstrated that gallium-68 fibroblast-activating protein inhibitor (^68^Ga-FAPI) PET/CT and ^18^F-FDG PET/CT yield differing results in detecting PBML [37,38]. The highly increased uptake of ^68^Ga-FAPI in PBML lesions suggests a high presence of activated fibroblasts [37]. Consequently, ^68^Ga-FAPI PET/CT could prove to have potential utility for the thorough assessment of PBML using imaging modalities [37,38].

VATS or open lung biopsy is considered the gold standard method for diagnosing PBML. CTPB can serve as an alternative for obtaining pathological specimens. However, as reported in Case 7, the patient initially underwent a CTPB at another institution, but the limited tissue sample was insufficient for a definitive diagnosis.

Histopathologically, PBML is characterized by well-circumscribed nodules with a firm consistency and an absence of necrosis or hemorrhage [23]. Uterine leiomyomas are characterized by spiral formations of benign smooth muscle cells. The immunohistochemical features of benign metastatic leiomyomas closely resemble those of uterine leiomyomas [23]. Positive markers include SMA, Desmin, ER, and PR, with a low Ki-67 score. Negative markers include S-100, CD34, and CD117 [2,11]. However, it has been reported that ER and PR may be negative in some cases [3]. In our case series, all patients tested positive for SMA and Desmin. ER expression was positive in five cases, weakly positive in one case, and negative in one case. PR expression was positive in four cases, weakly positive in one case, and not assessed in two cases.

Differential diagnosis of PBML includes a variety of benign and malignant pulmonary lesions that may share overlapping clinical, radiological, or histopathological features [39,40]. One of the most important differential diagnoses is lymphangioleiomyomatosis (LAM), which also affects women of reproductive age and is characterized by diffuse cystic lung lesions with abnormal smooth muscle-like cell proliferation along lymphatic vessels, blood vessels, and bronchi [6,41]. In contrast to PBML, which typically presents as well-defined solid pulmonary nodules, LAM demonstrates thin-walled cysts on imaging and expresses HMB-45 and melan-A on immunohistochemistry, while PBML is typically negative [6,42]. Another important consideration is pulmonary leiomyosarcoma, which should be suspected when histologic examination reveals cytologic atypia, high mitotic activity, and tumor necrosis [43,44]. Additionally, pulmonary hamartoma and other spindle cell tumors such as metastatic melanoma, malignant peripheral nerve sheath tumor, and inflammatory myofibroblastic tumor may also mimic PBML histologically [39,45]. A definitive diagnosis of PBML requires a comprehensive assessment incorporating the patient’s clinical and surgical history, characteristic imaging findings, histopathological morphology, and a panel of immunohistochemical markers.

### 4.3. Treatment and Prognosis

Due to the absence of randomized clinical trials, there are currently no established treatments available to guide the optimal management of PBML. According to the literature, treatments for PBML include hormone therapy, chemotherapy, and surgical resection [5,16,46,47,48].

Hormone therapy may be considered due to the positive expression of ER and PR in both primary tumors and metastatic lesions [5,47,48]. This therapy includes the use of gonadotropin-releasing hormone agonists, progesterone, and selective estrogen receptor modulators or aromatase inhibitors, which can help reduce estrogen stimulation [46,47,48]. The therapeutic effects of chemotherapy on patients with PBML are not completely consistent, and further investigation is still required [49,50,51]. Surgical treatment options include hysterectomy or oophorectomy, along with surgical resection of metastatic sites [5,46]. Bilateral oophorectomy decreases the secretion of estrogen and progesterone, resulting in therapeutic benefits [5,46]. For patients with solitary pulmonary nodules or a limited number of lesions, surgical resection of the pulmonary lesions combined with adjuvant anti-estrogen therapy may lead to favorable prognostic outcomes [23,25,52]. However, the side effects of estrogen deficiency can reduce quality of life and may even increase morbidity and mortality, necessitating close monitoring of the patient’s condition and careful clinical evaluation [53]. Asymptomatic patients with a stable clinical course may not need immediate treatment [2].

Treatment for each patient should be individualized, taking into account factors such as age, fertility status, hormonal profile, comorbidities, and presenting symptoms [6,25,47,49]. Due to its benign nature, PBML generally has a favorable prognosis. However, the potential for recurrence and malignant transformation exists, necessitating long-term surveillance [3,54]. In our study, Case 1 had previously undergone a total hysterectomy with left salpingo-oophorectomy. Due to the presence of multiple bilateral pulmonary nodules, she subsequently underwent right salpingo-oophorectomy after VATS. However, pelvic recurrence and inferior vena cava metastasis occurred three years after surgery. Case 6 did not undergo VATS and received treatment with GnRH agonists after CTPB. The remaining patients opted for regular surveillance without adjuvant therapy following VATS. All treatment decisions were made jointly by the patients and their families after receiving professional recommendations from the physicians. As of the last follow-up, all patients except Case 1 remained free of recurrence or metastasis and continue to receive routine outpatient monitoring.

Due to the rarity of PBML, particularly cases with coexisting in situ mucinous adenocarcinoma, it is inherently difficult to accumulate a large number of cases. This rarity naturally limits the sample size in our study and precludes statistical analysis. Nevertheless, a descriptive approach remains valuable for characterizing the clinical, radiological, and pathological features of such uncommon cases. Despite its limitations, our case series provides meaningful insight into the spectrum of PBML and highlights the importance of considering potential coexisting malignancies. In the future, multi-institutional collaborations may help to pool cases and allow for more robust statistical analyses. Furthermore, molecular and genetic studies could offer deeper insight into the pathogenesis, metastatic mechanisms, and potential malignant transformation of PBML, especially in patients with unusual histologic findings.

## 5. Conclusions

PBML is a rare condition that typically affects women with a history of uterine leiomyoma and usually exhibits indolent clinical behavior. In this retrospective case series of seven patients, PBML most commonly presented as bilateral or solitary pulmonary nodules, characterized by low proliferative activity and positivity for hormone receptors. Accurate diagnosis relied on imaging, histopathology, and immunohistochemistry, with VATS providing definitive diagnosis and therapeutic benefit in most cases. While most patients achieved favorable outcomes without adjuvant therapy, one patient experienced recurrence at an extrapulmonary site. Notably, we report the coexistence of intratumoral in situ mucinous adenocarcinoma within PBML, expanding the known histopathological spectrum of this entity and highlighting the importance of thorough pathological evaluation. Given the potential for recurrence or progression, continued surveillance and individualized management are essential. Due to the small sample size, these findings should be interpreted with caution, and further multicenter studies are warranted to better understand the clinical course and optimal treatment of PBML.

## Figures and Tables

**Table 1:** Clinical features and therapeutic outcomes.

Case	Age (Years)	Symptoms	Tumor Position	Max. Tumor Diameter (mm)	Active Surveillance Period	Gynecological Surgery	Time Since Hysterectomy (Years)	Diagnostic Method	Concurrent Tumors	Adjuvant Therapy	Follow-Up (Months)	Recurrence/Metastasis
1	40	Cough and sputum	Bilateral	21	7 months	Total hysterectomy + right adnexectomy	1.5	VATS	None	Left adnexectomy	36	Pelvic and IVC metastasis
2	52	No symptoms	RLL	9	1 week	Total hysterectomy + bilateral adnexectomy	8	VATS	None	None	44	None
3	51	No symptoms	RLL	10	1 month	None	None	VATS	None	None	36	None
4	47	No symptoms	Bilateral	13	1 month	None	None	VATS	None	None	20	None
5	58	No symptoms	RML	30	5 years	Total hysterectomy	16	VATS	In situ adenocarcinoma within PBML	None	16	None
6	47	No symptoms	Bilateral	28	2 weeks	Total hysterectomy	19	CT-guided percutaneous lung biopsy	None	GnRH	16	None
7	47	Cough and sputum	Bilateral	46	1 month	Total hysterectomy	21	VATS	None	None	14	None

VATS: video-assisted thoracic surgery; RLL: right lower lobe; RML: right middle lobe; IVC: inferior vena cava; GnRH: gonadotropin-releasing hormone; PBML: pulmonary benign metastasizing leiomyoma.

**Table 2:** Immunohistochemical analysis.

Case	VIM	SMA	Desmin	Caldesmon	ER	PR	Ki-67 LI
1	+	+	+	NA	+	+	1%
2	+	+	+	+	+	+	10%
3	NA	+	+	+	+	+	1%
4	NA	+	+	NA	Weak +	NA	<1%
5	NA	+	+	+	−	Weak +	3%
6	NA	+	+	+	+	NA	2%
7	NA	+	+	+	+	+	2%

VIM: Vimentin; SMA: smooth muscle actin; ER: estrogen receptor; PR: progesterone receptor; Ki-67 LI: Ki-67 labeling index; NA: not available; +: positive; −: negative.

## Data Availability

The original contributions presented in this study are included in the article. Further inquiries can be directed to the corresponding author.

## Abbreviations

The following abbreviations are used in this manuscript:

BML	Benign metastasizing leiomyoma
PBML	Pulmonary benign metastasizing leiomyoma
VATS	Video-assisted thoracoscopic surgery
CT	Computed tomography
CTPB	Computed tomography-guided percutaneous biopsy
ER	Estrogen receptor
PR	Progesterone receptor
SMA	Smooth muscle actin
PET/CT	Positron emission tomography–computed tomography
VIM	Vimentin
LI	Labeling index
FISH	Fluorescence in situ hybridization
GnRH	Gonadotropin-releasing hormone
MDT	Multidisciplinary team
^18^F-FDG	Fluorine-18 fluorodeoxyglucose
^68^Ga-FAPI	Gallium-68 fibroblast-activating protein inhibitor
